# *N*-Cyclo­hexyl-3-(4-hydr­oxy-6-oxo-1,6-dihydro­pyrimidin-5-yl)-3-*p*-tolyl­propanamide

**DOI:** 10.1107/S1600536809001251

**Published:** 2009-01-14

**Authors:** Xing-Han Wang, Wen-Juan Hao, Shu-Jiang Tu

**Affiliations:** a School of Chemistry and Chemical Engineering, Xuzhou Normal University, Xuzhou 221116, People’s Republic of China

## Abstract

In the mol­ecule of the title compound, C_20_H_25_N_3_O_3_, the aromatic rings are oriented at a dihedral angle of 88.36 (3)°. The cyclo­hexane ring adopts a chair conformation. In the crystal structure, inter­molecular N—H⋯O and O—H⋯N hydrogen bonds link the mol­ecules. C—H⋯π inter­actions are also present.

## Related literature

For general background, see: Johar *et al.* (2005 ▶); Janeba *et al.* (2005 ▶); Soloducho *et al.* (2003 ▶); Mathews & Asokan (2007 ▶); Lagoja (2005 ▶); Michael (2005 ▶); Erian (1993 ▶). For bond-length data, see: Allen *et al.* (1987 ▶). For ring-puckering parameters, see: Cremer & Pople (1975 ▶).
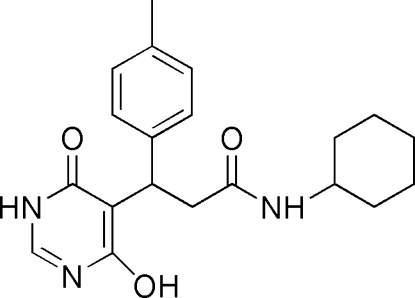


## Experimental

### 

#### Crystal data

C_20_H_25_N_3_O_3_
*M* *_r_* = 355.43
Monoclinic, 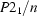

*a* = 7.1563 (12) Å
*b* = 19.637 (2) Å
*c* = 13.2746 (18) Å
β = 101.740 (2)°
*V* = 1826.5 (4) Å^3^
*Z* = 4
Mo *K*α radiation
μ = 0.09 mm^−1^
*T* = 298 (2) K
0.23 × 0.16 × 0.14 mm

#### Data collection

Bruker SMART CCD area-detector diffractometer
Absorption correction: multi-scan (*SADABS*; Bruker, 1998 ▶) *T*_min_ = 0.980, *T*_max_ = 0.988
9491 measured reflections
3216 independent reflections
1916 reflections with *I* > 2σ(*I*)
*R*_int_ = 0.073

#### Refinement

*R*[*F* ^2^ > 2σ(*F* ^2^)] = 0.048
*wR*(*F* ^2^) = 0.104
*S* = 1.02
3216 reflections
235 parameters
H-atom parameters constrained
Δρ_max_ = 0.16 e Å^−3^
Δρ_min_ = −0.19 e Å^−3^

### 

Data collection: *SMART* (Bruker, 1998 ▶); cell refinement: *SAINT* (Bruker, 1998 ▶); data reduction: *SAINT* (Bruker, 1998 ▶); program(s) used to solve structure: *SHELXS97* (Sheldrick, 2008 ▶); program(s) used to refine structure: *SHELXL97* (Sheldrick, 2008 ▶); molecular graphics: *SHELXTL* (Bruker, 1998 ▶); software used to prepare material for publication: *SHELXTL* (Bruker, 1998 ▶).

## Supplementary Material

Crystal structure: contains datablocks global, I. DOI: 10.1107/S1600536809001251/hk2610sup1.cif

Structure factors: contains datablocks I. DOI: 10.1107/S1600536809001251/hk2610Isup2.hkl

Additional supplementary materials:  crystallographic information; 3D view; checkCIF report

## Figures and Tables

**Table 1:** Hydrogen-bond geometry (Å, °)

*D*—H⋯*A*	*D*—H	H⋯*A*	*D*⋯*A*	*D*—H⋯*A*
N1—H1⋯O3^i^	0.86	1.98	2.813 (3)	162
O1—H1*A*⋯N2^ii^	0.82	1.95	2.753 (3)	168
N3—H3⋯O2^iii^	0.86	2.19	2.992 (4)	155
C17—H17*B*⋯*Cg*2^iv^	0.97	2.47	3.440 (3)	177
C20—H20*A*⋯*Cg*2^v^	0.97	2.74	3.629 (3)	152

Symmetry codes: (i) 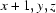; (ii) 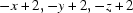; (iii) 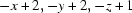; (iv) 
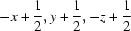
; (v) 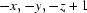. *Cg*2 is centroid of the C8–C13 ring.

**Fig. 1.**
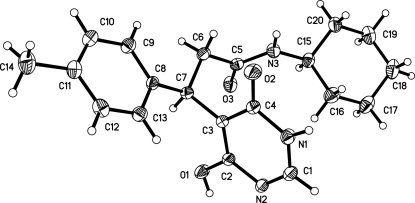
The molecular structure of the title molecule, with the atom-numbering scheme. Displacement ellipsoids are drawn at the 50% probability level.

## supplementary crystallographic information

### Comment

The pyrimidines and their derivatives as a class of extremely important heterocyclic compounds are used in a wide array of synthetic and industrial applications. Not only they are an integral part of the genetic materials, *viz*. DNA and RNA as nucleotides and nucleosides but also play critical roles especially in pharmaceutical fields (Johar *et al.*, 2005; Janeba *et al.*, 2005). Some pyrimidine derivatives can give stable and good quality nanomaterials having many important electrical and optical properties (Soloducho *et al.*, 2003; Mathews & Asokan, 2007), and also used as functional materials (Lagoja, 2005; Michael, 2005; Erian, 1993). We report herein the crystal structure of the title compound.

In the molecule of the title compound (Fig 1), the bond lengths (Allen *et al.*,  1987) and angles are within normal ranges. Rings A (N1/N2/C1-C4) and B (C8-C13) are, of course, planar, and they are oriented at a dihedral angle of 88.36 (3)°. The cyclohexane ring C (C15-C20), having total puckering amplitude, Q_T_, of 0.565 (3) Å, chair conformation [φ = -30.33 (3)° and θ = 4.00 (3)°] (Cremer & Pople, 1975).

In the crystal structure, intermolecular N-H···O and O-H···N hydrogen bonds (Table 1) link the molecules, in which they may be effective in the stabilization of the structure. There also exist C–H···π interactions (Table 1).

### Experimental

The title compound was prepared by the reaction of *p*-tolylidene-Meldrum's acid (1 mmol) with 6-hydroxypyrimidin-4(3*H*)-one (1 mmol) and cyclohexanamine (1 mmol) at 373 K in glacial acetic acid under microwave irradiation (maximum power 250 W, initial power 100 W) for 18 min (yield; 83%, m.p. 534–536 K). Crystals suitable for X-ray analysis were obtained from an ethanol solution by slow evaporation.

### Refinement

H atoms were positioned geometrically, with O-H = 0.82 Å (for OH), N-H = 0.86 Å (for NH) and C-H = 0.93, 0.98, 0.97 and 0.96 Å for aromatic, methine, methylene and methyl H, respectively, and constrained to ride on their parent atoms, with U_iso_(H) = xU_eq_(C,N,O), where x = 1.5 for methyl and OH H and x = 1.2 for all other H atoms.

### Crystal data

**Table:**

C_20_H_25_N_3_O_3_	*F*(000) = 760
*M**_r_* = 355.43	*D*_x_ = 1.293 Mg m^−^^3^
Monoclinic, *P*2_1_/*n*	Melting point = 534–536 K
Hall symbol: -P 2yn	Mo *K*α radiation, λ = 0.71073 Å
*a* = 7.1563 (12) Å	Cell parameters from 1921 reflections
*b* = 19.637 (2) Å	θ = 2.6–27.7°
*c* = 13.2746 (18) Å	µ = 0.09 mm^−^^1^
β = 101.740 (2)°	*T* = 298 K
*V* = 1826.5 (4) Å^3^	Block, colorless
*Z* = 4	0.23 × 0.16 × 0.14 mm

### Data collection

**Table:**

Bruker SMART CCD area-detector diffractometer	3216 independent reflections
Radiation source: fine-focus sealed tube	1916 reflections with *I* > 2σ(*I*)
graphite	*R*_int_ = 0.073
φ and ω scans	θ_max_ = 25.0°, θ_min_ = 1.9°
Absorption correction: multi-scan (*SADABS*; Bruker, 1998)	*h* = −8→8
*T*_min_ = 0.980, *T*_max_ = 0.988	*k* = −23→21
9491 measured reflections	*l* = −15→15

### Refinement

**Table:**

Refinement on *F*^2^	Primary atom site location: structure-invariant direct methods
Least-squares matrix: full	Secondary atom site location: difference Fourier map
*R*[*F*^2^ > 2σ(*F*^2^)] = 0.048	Hydrogen site location: inferred from neighbouring sites
*wR*(*F*^2^) = 0.104	H-atom parameters constrained
*S* = 1.02	*w* = 1/[σ^2^(*F*_o_^2^) + (0.0354*P*)^2^] where *P* = (*F*_o_^2^ + 2*F*_c_^2^)/3
3216 reflections	(Δ/σ)_max_ = 0.001
235 parameters	Δρ_max_ = 0.16 e Å^−^^3^
0 restraints	Δρ_min_ = −0.19 e Å^−^^3^

### Special details

**Table:**

Geometry. All e.s.d.'s (except the e.s.d. in the dihedral angle between two l.s. planes) are estimated using the full covariance matrix. The cell e.s.d.'s are taken into account individually in the estimation of e.s.d.'s in distances, angles and torsion angles; correlations between e.s.d.'s in cell parameters are only used when they are defined by crystal symmetry. An approximate (isotropic) treatment of cell e.s.d.'s is used for estimating e.s.d.'s involving l.s. planes.
Refinement. Refinement of *F*^2^ against ALL reflections. The weighted *R*-factor *wR* and goodness of fit *S* are based on *F*^2^, conventional *R*-factors *R* are based on *F*, with *F* set to zero for negative *F*^2^. The threshold expression of *F*^2^ > σ(*F*^2^) is used only for calculating *R*-factors(gt) *etc*. and is not relevant to the choice of reflections for refinement. *R*-factors based on *F*^2^ are statistically about twice as large as those based on *F*, and *R*- factors based on ALL data will be even larger.

### Fractional atomic coordinates and isotropic or equivalent isotropic displacement parameters (Å^2^)

**Table:**

	*x*	*y*	*z*	*U*_iso_*/*U*_eq_	
O1	0.8327 (2)	1.04303 (8)	0.89288 (11)	0.0456 (5)	
H1A	0.8598	1.0297	0.9526	0.068*	
O2	1.1335 (2)	1.04358 (9)	0.60752 (11)	0.0511 (5)	
O3	0.5892 (2)	0.93937 (8)	0.69802 (11)	0.0447 (4)	
N1	1.2782 (3)	1.00038 (10)	0.76292 (14)	0.0387 (5)	
H1	1.3786	0.9909	0.7394	0.046*	
N2	1.1340 (3)	0.99789 (9)	0.90587 (13)	0.0367 (5)	
N3	0.7956 (3)	0.91122 (9)	0.59665 (13)	0.0378 (5)	
H3	0.8514	0.9251	0.5488	0.045*	
C1	1.2753 (3)	0.98462 (12)	0.86043 (17)	0.0396 (6)	
H1B	1.3815	0.9626	0.8987	0.048*	
C2	0.9778 (3)	1.02944 (11)	0.84599 (16)	0.0316 (5)	
C3	0.9650 (3)	1.04690 (10)	0.74467 (15)	0.0290 (5)	
C4	1.1234 (3)	1.03189 (11)	0.69730 (17)	0.0346 (6)	
C5	0.6857 (3)	0.95565 (11)	0.63376 (16)	0.0329 (6)	
C6	0.6872 (3)	1.02749 (11)	0.59477 (16)	0.0350 (6)	
H6A	0.5572	1.0422	0.5679	0.042*	
H6B	0.7563	1.0291	0.5391	0.042*	
C7	0.7820 (3)	1.07626 (11)	0.68124 (15)	0.0317 (6)	
H7	0.6931	1.0795	0.7283	0.038*	
C8	0.8005 (3)	1.14835 (11)	0.64204 (16)	0.0302 (5)	
C9	0.6815 (3)	1.17248 (12)	0.55327 (17)	0.0366 (6)	
H9	0.6022	1.1421	0.5108	0.044*	
C10	0.6781 (3)	1.24068 (12)	0.52658 (18)	0.0400 (6)	
H10	0.5952	1.2553	0.4672	0.048*	
C11	0.7955 (4)	1.28759 (12)	0.58638 (19)	0.0393 (6)	
C12	0.9218 (4)	1.26311 (12)	0.67173 (19)	0.0412 (6)	
H12	1.0068	1.2931	0.7115	0.049*	
C13	0.9251 (3)	1.19530 (12)	0.69940 (17)	0.0367 (6)	
H13	1.0118	1.1806	0.7573	0.044*	
C14	0.7807 (4)	1.36269 (12)	0.5595 (2)	0.0587 (8)	
H14A	0.8643	1.3731	0.5135	0.088*	
H14B	0.8168	1.3892	0.6212	0.088*	
H14C	0.6517	1.3734	0.5269	0.088*	
C15	0.8280 (3)	0.84081 (11)	0.63122 (17)	0.0367 (6)	
H15	0.7200	0.8266	0.6614	0.044*	
C16	1.0092 (4)	0.83383 (13)	0.71338 (18)	0.0488 (7)	
H16A	0.9999	0.8627	0.7714	0.059*	
H16B	1.1174	0.8492	0.6857	0.059*	
C17	1.0429 (4)	0.76056 (13)	0.75025 (19)	0.0532 (7)	
H17A	1.1649	0.7576	0.7979	0.064*	
H17B	0.9442	0.7473	0.7868	0.064*	
C18	1.0418 (4)	0.71171 (14)	0.6614 (2)	0.0593 (8)	
H18A	1.1538	0.7198	0.6324	0.071*	
H18B	1.0483	0.6653	0.6869	0.071*	
C19	0.8642 (4)	0.72005 (12)	0.5780 (2)	0.0556 (8)	
H19A	0.7536	0.7057	0.6043	0.067*	
H19B	0.8738	0.6910	0.5202	0.067*	
C20	0.8367 (4)	0.79365 (12)	0.54125 (18)	0.0435 (6)	
H20A	0.9418	0.8070	0.5095	0.052*	
H20B	0.7195	0.7975	0.4900	0.052*	

### Atomic displacement parameters (Å^2^)

**Table:**

	*U*^11^	*U*^22^	*U*^33^	*U*^12^	*U*^13^	*U*^23^
O1	0.0413 (10)	0.0669 (13)	0.0301 (9)	0.0186 (9)	0.0109 (8)	0.0084 (8)
O2	0.0557 (12)	0.0680 (13)	0.0339 (10)	0.0139 (10)	0.0193 (9)	0.0113 (8)
O3	0.0467 (11)	0.0426 (10)	0.0503 (10)	0.0067 (8)	0.0227 (9)	0.0080 (8)
N1	0.0312 (12)	0.0497 (13)	0.0373 (11)	0.0088 (10)	0.0120 (10)	0.0044 (10)
N2	0.0357 (12)	0.0444 (13)	0.0295 (11)	0.0122 (10)	0.0057 (9)	0.0041 (9)
N3	0.0432 (13)	0.0354 (12)	0.0390 (11)	0.0032 (10)	0.0185 (10)	0.0063 (9)
C1	0.0356 (15)	0.0456 (15)	0.0353 (14)	0.0085 (12)	0.0020 (12)	0.0041 (11)
C2	0.0292 (13)	0.0365 (14)	0.0286 (13)	0.0043 (11)	0.0047 (11)	−0.0004 (10)
C3	0.0301 (13)	0.0301 (13)	0.0257 (12)	0.0031 (11)	0.0030 (10)	0.0007 (10)
C4	0.0371 (15)	0.0360 (14)	0.0304 (13)	0.0042 (12)	0.0061 (12)	0.0026 (11)
C5	0.0296 (14)	0.0373 (15)	0.0302 (13)	−0.0008 (11)	0.0024 (11)	0.0011 (11)
C6	0.0363 (15)	0.0344 (14)	0.0319 (13)	0.0028 (11)	0.0016 (11)	0.0038 (10)
C7	0.0340 (14)	0.0347 (14)	0.0263 (12)	0.0042 (11)	0.0059 (11)	0.0018 (10)
C8	0.0277 (13)	0.0324 (14)	0.0305 (13)	0.0052 (11)	0.0063 (11)	0.0021 (11)
C9	0.0315 (14)	0.0359 (15)	0.0403 (14)	−0.0024 (11)	0.0020 (12)	0.0036 (11)
C10	0.0372 (15)	0.0397 (15)	0.0420 (15)	0.0012 (13)	0.0053 (12)	0.0081 (12)
C11	0.0348 (15)	0.0356 (15)	0.0493 (16)	0.0008 (12)	0.0130 (13)	0.0031 (12)
C12	0.0328 (15)	0.0378 (15)	0.0537 (17)	−0.0081 (12)	0.0104 (13)	−0.0084 (12)
C13	0.0292 (14)	0.0418 (15)	0.0376 (14)	0.0014 (12)	0.0034 (12)	−0.0012 (11)
C14	0.066 (2)	0.0381 (17)	0.0760 (19)	0.0006 (14)	0.0241 (16)	0.0061 (14)
C15	0.0355 (15)	0.0356 (15)	0.0413 (14)	0.0049 (11)	0.0132 (12)	0.0074 (11)
C16	0.0460 (17)	0.0539 (17)	0.0462 (16)	0.0012 (14)	0.0090 (14)	0.0077 (13)
C17	0.0423 (17)	0.0642 (19)	0.0551 (17)	0.0101 (14)	0.0148 (14)	0.0224 (15)
C18	0.0567 (19)	0.0523 (19)	0.075 (2)	0.0196 (15)	0.0281 (17)	0.0207 (15)
C19	0.059 (2)	0.0414 (17)	0.071 (2)	0.0062 (14)	0.0233 (17)	−0.0026 (14)
C20	0.0406 (16)	0.0427 (16)	0.0477 (15)	0.0063 (13)	0.0099 (13)	−0.0021 (12)

### Geometric parameters (Å, °)

**Table:**

N1—C1	1.335 (3)	C10—H10	0.9300
N1—C4	1.406 (3)	C11—C12	1.384 (3)
N1—H1	0.8600	C11—C14	1.516 (3)
N2—C1	1.305 (3)	C12—C13	1.380 (3)
N2—C2	1.380 (3)	C12—H12	0.9300
N3—C5	1.334 (3)	C13—H13	0.9300
N3—C15	1.460 (3)	C14—H14A	0.9600
N3—H3	0.8600	C14—H14B	0.9600
O1—C2	1.341 (2)	C14—H14C	0.9600
O1—H1A	0.8200	C15—C16	1.522 (3)
O2—C4	1.230 (2)	C15—C20	1.523 (3)
O3—C5	1.244 (2)	C15—H15	0.9800
C1—H1B	0.9300	C16—C17	1.523 (3)
C2—C3	1.373 (3)	C16—H16A	0.9700
C3—C4	1.435 (3)	C16—H16B	0.9700
C3—C7	1.519 (3)	C17—C18	1.519 (4)
C5—C6	1.504 (3)	C17—H17A	0.9700
C6—C7	1.542 (3)	C17—H17B	0.9700
C6—H6A	0.9700	C18—C19	1.515 (4)
C6—H6B	0.9700	C18—H18A	0.9700
C7—C8	1.523 (3)	C18—H18B	0.9700
C7—H7	0.9800	C19—C20	1.525 (3)
C8—C9	1.390 (3)	C19—H19A	0.9700
C8—C13	1.396 (3)	C19—H19B	0.9700
C9—C10	1.384 (3)	C20—H20A	0.9700
C9—H9	0.9300	C20—H20B	0.9700
C10—C11	1.383 (3)		
			
C1—N1—C4	122.5 (2)	C13—C12—H12	119.1
C1—N1—H1	118.8	C11—C12—H12	119.1
C4—N1—H1	118.8	C12—C13—C8	121.2 (2)
C1—N2—C2	115.89 (18)	C12—C13—H13	119.4
C5—N3—C15	124.82 (18)	C8—C13—H13	119.4
C5—N3—H3	117.6	C11—C14—H14A	109.5
C15—N3—H3	117.6	C11—C14—H14B	109.5
C2—O1—H1A	109.5	H14A—C14—H14B	109.5
N2—C1—N1	124.6 (2)	C11—C14—H14C	109.5
N2—C1—H1B	117.7	H14A—C14—H14C	109.5
N1—C1—H1B	117.7	H14B—C14—H14C	109.5
O1—C2—C3	120.1 (2)	N3—C15—C16	111.59 (19)
O1—C2—N2	115.78 (18)	N3—C15—C20	111.02 (18)
C3—C2—N2	124.1 (2)	C16—C15—C20	110.01 (19)
C2—C3—C4	118.5 (2)	N3—C15—H15	108.0
C2—C3—C7	121.06 (19)	C16—C15—H15	108.0
C4—C3—C7	120.26 (18)	C20—C15—H15	108.0
O2—C4—N1	119.2 (2)	C15—C16—C17	111.8 (2)
O2—C4—C3	126.4 (2)	C15—C16—H16A	109.3
N1—C4—C3	114.34 (18)	C17—C16—H16A	109.3
O3—C5—N3	122.4 (2)	C15—C16—H16B	109.3
O3—C5—C6	121.4 (2)	C17—C16—H16B	109.3
N3—C5—C6	116.14 (19)	H16A—C16—H16B	107.9
C5—C6—C7	111.05 (17)	C18—C17—C16	111.8 (2)
C5—C6—H6A	109.4	C18—C17—H17A	109.3
C7—C6—H6A	109.4	C16—C17—H17A	109.3
C5—C6—H6B	109.4	C18—C17—H17B	109.3
C7—C6—H6B	109.4	C16—C17—H17B	109.3
H6A—C6—H6B	108.0	H17A—C17—H17B	107.9
C3—C7—C8	114.65 (18)	C19—C18—C17	111.7 (2)
C3—C7—C6	112.09 (17)	C19—C18—H18A	109.3
C8—C7—C6	112.24 (17)	C17—C18—H18A	109.3
C3—C7—H7	105.7	C19—C18—H18B	109.3
C8—C7—H7	105.7	C17—C18—H18B	109.3
C6—C7—H7	105.7	H18A—C18—H18B	107.9
C9—C8—C13	116.8 (2)	C18—C19—C20	111.7 (2)
C9—C8—C7	121.7 (2)	C18—C19—H19A	109.3
C13—C8—C7	121.2 (2)	C20—C19—H19A	109.3
C10—C9—C8	121.5 (2)	C18—C19—H19B	109.3
C10—C9—H9	119.2	C20—C19—H19B	109.3
C8—C9—H9	119.2	H19A—C19—H19B	107.9
C11—C10—C9	121.4 (2)	C15—C20—C19	110.4 (2)
C11—C10—H10	119.3	C15—C20—H20A	109.6
C9—C10—H10	119.3	C19—C20—H20A	109.6
C10—C11—C12	117.3 (2)	C15—C20—H20B	109.6
C10—C11—C14	120.5 (2)	C19—C20—H20B	109.6
C12—C11—C14	122.2 (2)	H20A—C20—H20B	108.1
C13—C12—C11	121.7 (2)		
			
C2—N2—C1—N1	1.1 (3)	C3—C7—C8—C9	153.35 (19)
C4—N1—C1—N2	−1.8 (4)	C6—C7—C8—C9	24.0 (3)
C1—N2—C2—O1	180.0 (2)	C3—C7—C8—C13	−33.2 (3)
C1—N2—C2—C3	−0.1 (3)	C6—C7—C8—C13	−162.62 (19)
O1—C2—C3—C4	179.7 (2)	C13—C8—C9—C10	−4.0 (3)
N2—C2—C3—C4	−0.3 (3)	C7—C8—C9—C10	169.7 (2)
O1—C2—C3—C7	−5.2 (3)	C8—C9—C10—C11	1.0 (3)
N2—C2—C3—C7	174.9 (2)	C9—C10—C11—C12	2.5 (3)
C1—N1—C4—O2	−178.3 (2)	C9—C10—C11—C14	−176.1 (2)
C1—N1—C4—C3	1.3 (3)	C10—C11—C12—C13	−3.1 (3)
C2—C3—C4—O2	179.2 (2)	C14—C11—C12—C13	175.5 (2)
C7—C3—C4—O2	4.0 (4)	C11—C12—C13—C8	0.1 (3)
C2—C3—C4—N1	−0.3 (3)	C9—C8—C13—C12	3.4 (3)
C7—C3—C4—N1	−175.56 (18)	C7—C8—C13—C12	−170.3 (2)
C15—N3—C5—O3	−4.8 (3)	C5—N3—C15—C16	−94.9 (3)
C15—N3—C5—C6	174.2 (2)	C5—N3—C15—C20	142.0 (2)
O3—C5—C6—C7	66.6 (3)	N3—C15—C16—C17	179.60 (18)
N3—C5—C6—C7	−112.4 (2)	C20—C15—C16—C17	−56.7 (3)
C2—C3—C7—C8	117.4 (2)	C15—C16—C17—C18	54.0 (3)
C4—C3—C7—C8	−67.5 (3)	C16—C17—C18—C19	−52.2 (3)
C2—C3—C7—C6	−113.1 (2)	C17—C18—C19—C20	54.0 (3)
C4—C3—C7—C6	62.0 (3)	N3—C15—C20—C19	−178.2 (2)
C5—C6—C7—C3	44.5 (2)	C16—C15—C20—C19	57.8 (3)
C5—C6—C7—C8	175.24 (18)	C18—C19—C20—C15	−57.0 (3)

### Hydrogen-bond geometry (Å, °)

**Table:**

*D*—H···*A*	*D*—H	H···*A*	*D*···*A*	*D*—H···*A*
N1—H1···O3^i^	0.86	1.98	2.813 (3)	162
O1—H1A···N2^ii^	0.82	1.95	2.753 (3)	168
N3—H3···O2^iii^	0.86	2.19	2.992 (4)	155
C17—H17B···Cg2^iv^	0.97	2.47	3.440 (3)	177
C20—H20A···Cg2^v^	0.97	2.74	3.629 (3)	152

Symmetry codes: (i) *x*+1, *y*, *z*; (ii) −*x*+2, −*y*+2, −*z*+2; (iii) −*x*+2, −*y*+2, −*z*+1; (iv) −*x*+1/2, *y*+1/2, −*z*+1/2; (v) −*x*, −*y*, −*z*+1.

## References

[bb1] Allen, F. H., Kennard, O., Watson, D. G., Brammer, L., Orpen, A. G. & Taylor, R. (1987). *J. Chem. Soc. Perkin Trans. 2*, pp. S1–19.

[bb2] Bruker (1998). *SMART*, *SAINT* and *SADABS* Bruker AXS Inc., Madison, Wisconsin, USA.

[bb3] Cremer, D. & Pople, J. A. (1975). *J. Am. Chem. Soc.* **97**, 1354–1358.

[bb4] Erian, A. W. (1993). *Chem. Rev.* **93**, 1991–2005.

[bb5] Janeba, Z., Balzarini, J., Andrei, G., Snoeck, R., De Clercq, E. & Robins, M. J. (2005). *J. Med. Chem.* **48**, 4690–4696. 10.1021/jm050291s 16000005

[bb6] Johar, M., Manning, T., Kunimoto, D. Y. & Kumar, R. (2005). *Bioorg. Med. Chem.* **13**, 6663–6671. 10.1016/j.bmc.2005.07.046 16140016

[bb7] Lagoja, I. M. (2005). *Chem. Biodivers.* **2**, 1–50. 10.1002/cbdv.200490173 17191918

[bb8] Mathews, A. & Asokan, C. V. (2007). Tetrahedron, 63, 7845-7849.

[bb9] Michael, J. P. (2005). *Nat. Prod. Rep.* **22**, 627 646. 10.1039/b413750g 16193160

[bb10] Sheldrick, G. M. (2008). *Acta Cryst.* A**64**, 112–122. 10.1107/S0108767307043930 18156677

[bb11] Soloducho, J., Doskocz, J., Cabaj, J. & Roszak, S. (2003). *Tetrahedron*, **59**, 4761–4766.

